# Altered Brain Connectivity in Early Postmenopausal Women with Subjective Cognitive Impairment

**DOI:** 10.3389/fnins.2016.00433

**Published:** 2016-09-23

**Authors:** Jennifer N. Vega, Lilia Zurkovsky, Kimberly Albert, Alyssa Melo, Brian Boyd, Julie Dumas, Neil Woodward, Brenna C. McDonald, Andrew J. Saykin, Joon H. Park, Magdalena Naylor, Paul A. Newhouse

**Affiliations:** ^1^Department of Psychiatry, Center for Cognitive Medicine, Vanderbilt University School of MedicineNashville, TN, USA; ^2^Clinical Neuroscience Research Unit, Department of Psychiatry, University of Vermont College of MedicineBurlington, VT, USA; ^3^Department of Radiology and Imaging Sciences and the Indiana Alzheimer Disease Center, Indiana University School of MedicineIndianapolis, IN, USA; ^4^Department of Psychiatry, Jeju National University School of MedicineJejudo, South Korea; ^5^Department of Veterans Affairs, Geriatric Research, Education, and Clinical Center, Tennessee Valley Health SystemNashville, TN, USA

**Keywords:** resting state fMRI, functional connectivity, post-menopausal women, subjective cognitive complaints, subjective cognitive impairment

## Abstract

Cognitive changes after menopause are a common complaint, especially as the loss of estradiol at menopause has been hypothesized to contribute to the higher rates of dementia in women. To explore the neural processes related to subjective cognitive complaints, this study examined resting state functional connectivity in 31 postmenopausal women (aged 50–60) in relationship to cognitive complaints following menopause. A cognitive complaint index was calculated using responses to a 120-item questionnaire. Seed regions were identified for resting state brain networks important for higher-order cognitive processes and for areas that have shown differences in volume and functional activity associated with cognitive complaints in prior studies. Results indicated a positive correlation between the executive control network and cognitive complaint score, weaker negative functional connectivity within the frontal cortex, and stronger positive connectivity within the right middle temporal gyrus in postmenopausal women who report more cognitive complaints. While longitudinal studies are needed to confirm this hypothesis, these data are consistent with previous findings suggesting that high levels of cognitive complaints may reflect changes in brain connectivity and may be a potential marker for the risk of late-life cognitive dysfunction in postmenopausal women with otherwise normal cognitive performance.

## Introduction

The perception of a change in cognitive or memory abilities is common in aging. Prior to the last decade, the meaning of subjective cognitive complaints has been unclear, as subjective cognitive complaints have not correlated well with objective decline in performance on standardized neuropsychological assessment (Mendes et al., 2008). However, there is increasing evidence to support that reports of subjective cognitive decline (SCD), even with normal performance on objective cognitive tests, is associated with increased likelihood of Alzheimer disease (AD) biomarker abnormalities and with an increased risk for future cognitive decline and AD (Jonker et al., 2000; Saykin et al., 2006; Visser et al., 2009; Jessen et al., 2010, 2014a; Reisberg et al., 2010; Rami et al., 2011; Amariglio et al., 2012; Wang et al., 2013; Vega and Newhouse, 2014). Although, SCD is non-specific and could potentially reflect numerous conditions such as normal aging, psychiatric conditions, neurologic and medical disorders, substance use, and medication effects (Kim et al., 2003; Reid and Maclullich, 2006; Elfgren et al., 2010; Slavin et al., 2010; Bartley et al., 2012; Vega and Newhouse, 2014), current evidence suggests that subjective memory complaints may be a predictor of later development of mild cognitive impairment (MCI) and dementia (Geerlings et al., 1999; Palmer et al., 2003; van Oijen et al., 2007; Reisberg et al., 2010; Waldorff et al., 2012; Genziani et al., 2013).

Several neuroimaging studies have found structural and functional brain differences in individuals with and without subjective cognitive complaints. Cross-sectional studies have shown reduced hippocampal volume in older adults with subjective memory complaints but normal performance on objective memory tests compared to individuals without subjective memory complaints (Saykin et al., 2006). Jessen and colleagues also showed reduction in entorhinal cortex volume in individuals with subjective memory complaints that was intermediate between healthy controls and patients with MCI (Jessen et al., 2007). Longitudinal studies have confirmed these findings and shown that subjective memory complaints have predicted volume decline in gray matter, particularly in medial temporal lobe structures (Stewart et al., 2011; Scheef et al., 2012; Hafkemeijer et al., 2013; Cherbuin et al., 2015). Glucose metabolism as measured by FDG-PET was shown to be altered in individuals with subjective memory complaints compared to controls (Scheef et al., 2012), and Rodda and colleagues found increased brain activation (BOLD signal) in task relevant brain regions in both encoding and attention tasks despite normal performance in older adults (Rodda et al., 2009, 2011). A longitudinal study found that subjective cognitive complaints not only predicted a decline in cognitive performance but also correlated with increased cerebral blood flow as measured by FDG-PET during a memory task (Hohman et al., 2011).

There is evidence to suggest subjective cognitive complaints are more common and may be more predictive of later cognitive dysfunction in women (Pérès et al., 2011). Memory complaints during or after the menopause transition are common. Approximately 60% of middle-aged women reported cognitive changes in the Seattle Midlife Women's Health Study (Sullivan and Woods, 2001), and 42% of postmenopausal women reported a negative change in cognition in the Study of Women Across the Nation (SWAN) (Bromberger et al., 2011). In addition to increased cognitive complaints during or after menopause, women also appear to be at higher risk for AD, particularly if they carry the *APOE*ε*4* allele (Bretsky et al., 1999). Studies that have examined objective cognitive performance in relation to the menopause transition have had mixed results (Hogervorst and Bandelow, 2010; Dumas et al., 2013). One study showed that subjective cognitive complaints were associated with menopause-related physical symptoms, psychological factors, and objective impairment (Schaafsma et al., 2010). Other studies have shown that subtle deficits in objective cognitive performance correlated with some measures of poorer subjective memory performance (Drogos et al., 2013; Thurston, 2013). Subjective memory complaints in perimenopausal women were found to be most associated with working memory and complex attention rather than verbal episodic learning or memory (Weber et al., 2012) suggesting that high effort demanding cognitive operations may lead to the perception of subjective cognitive difficulties. In a study of working memory examining a subset of participants used in the current study, Dumas and colleagues (Dumas et al., 2013) found that women with substantial postmenopausal cognitive complaints showed greater cortical activity (measured via BOLD signal) during working memory performance than women without such complaints despite equivalent performance, suggesting that cognitive complaints may indicate increased neural effort, perhaps as a form of compensation.

In addition to task related activity, resting state functional connectivity (rsFC) has been associated with measures of cognitive performance and behavioral state (Hampson et al., 2006; Keller et al., 2015). rsFC is a technique that can be used to identify brain networks by measuring the temporal correlations of low-frequency (<0.1 Hz) BOLD signal fluctuations between brain regions while the brain is at rest (i.e., not actively performing a goal-directed task). These low-frequency fluctuations are thought to reflect the intrinsic functional architecture of the brain (Fox and Raichle, 2007). rsFC has revealed that signals in functionally related brain regions correlate with each other even in the absence of external stimuli (Greicius et al., 2003). Differences in the connectivity of these functionally related brain regions can reflect changes in structural connectivity or efficiency of constituent brain circuits (Sheline and Raichle, 2013). rsFC studies have identified numerous brain networks, the most commonly investigated of which is the default mode network (DMN) (Raichle et al., 2001; Greicius et al., 2003). In addition to the DMN, rsFC studies have identified several other resting state brain networks associated with higher cognitive functioning (Yeo et al., 2011), such as the dorsal and ventral attention networks (DAN and VAN, respectively) (Fox et al., 2006), the salience network (Seeley et al., 2007), and the executive control network (ECN) (Seeley et al., 2007; Vincent et al., 2008). Connectivity strength of the DMN, ECN, Salience, and DAN have been associated with performance on cognitive tasks (van den Heuvel and Hulshoff Pol, 2010).

Recent studies have suggested that subjective memory complaints in the elderly may be associated with altered rsFC (Bajo et al., 2012; Hafkemeijer et al., 2013). Cognitive complaints after menopause may be a particularly meaningful marker for early neural dysfunction, especially as loss of estradiol at menopause has been hypothesized to contribute to the higher rates of dementia in women. To examine whether subjective cognitive complaints after menopause show a relationship to changes in functional connectivity, we examined rsFC in 31 middle-aged, post-menopausal women (aged 50–60) who were cognitively normal on objective tests, but self-reported an experience of cognitive decline since menopause. Since connectivity strength of the DMN, ECN, Salience, and DAN have been associated with performance on cognitive tasks in previous studies (van den Heuvel and Hulshoff Pol, 2010), those networks were included in the analysis. In addition to the functional connectivity networks chosen, additional seed regions that showed structural and functional differences between cognitive complainers and control groups were also included in the analysis (Saykin et al., 2006; Wang et al., 2006, 2013; Dumas et al., 2013). We hypothesized that postmenopausal women with significant subjective cognitive complaints would show increased functional connectivity in one or more resting state brain networks suggesting early neural compensation, potentially a harbinger of increased risk for late life cognitive dysfunction.

## Methods

### Participants

Participants were recruited through notices and advertisements in local newspapers and direct mailings. Participants were required to be postmenopausal (i.e., without menses for 1 year and without surgically induced menopause). Exclusion criteria for all participants included: (1) any active neurologic and/or psychiatric disease, history of significant head trauma followed by persistent neurologic deficits, or known structural brain abnormalities, (2) current major depression or another major psychiatric disorder as described in DSM-IV (use of psychotropic medications (e.g., antidepressants), (3) any history of alcohol or substance abuse or dependence, (4) any significant systemic illness or unstable medical condition which could lead to difficulty complying with the protocol including: (4a) history of myocardial infarction in the past year or unstable, severe cardiovascular disease including angina or CHF with symptoms at rest, or clinically significant abnormalities on the ECG (4b) clinically significant and/or unstable pulmonary, gastrointestinal, hepatic, or renal disease (4c) insulin-requiring diabetes or uncontrolled diabetes mellitus, (4d) uncontrolled hypertension (systolic BP > 160 or diastolic BP > 100), (5) use of hormone therapy during the last year, (6) a history of breast cancer, and (7) and a history or presence of severe menopausal symptoms. Exclusion criteria for MRI scanning included: (1) non-removable ferromagnetic material on or in the body and (2) claustrophobia.

A total of 53 healthy, post-menopausal were recruited and screened. Of this sample, 32 women completed a resting state scan; one participant was eliminated due to a brain abnormality. Data were analyzed with 31 participants. The current study was conducted at the University of Vermont (*n* = 11) and at Vanderbilt University (*n* = 20). Both University of Vermont and Vanderbilt University Institutional Review Boards approved all study protocols. This study was carried out in accordance with the recommendations of University of Vermont and Vanderbilt University Institutional Review Boards with written informed consent from all participants. All participants gave written informed consent in accordance with the Declaration of Helsinki.

### Cognitive and behavioral screening

Upon meeting inclusion and exclusion criteria, participants were approved for further cognitive and behavioral screening. After signing informed consent documents, participants gave a medical history and underwent a physical and laboratory tests assessing hematopoietic, renal, hepatic and hormonal function. Participants were cognitively screened using the Mini-Mental State Exam (MMSE; score ≥ 27; (Folstein et al., 1975), Brief Cognitive Rating Scale (score ≤ 2) (Reisberg et al., 1988) and Mattis Dementia Rating Scale (DRS; minimum score 123) (Jurica et al., 2001) to establish a Global Deterioration Scale score (GDS; score ≤ 2) (Reisberg et al., 1993) which rates the degree of cognitive impairment. Behavioral screening consisted of a partial Structured Clinical Interview (SCID) for DSM disorders (First et al., 2002) and the Beck Depression Inventory (BDI; score ≤ 7) (Beck et al., 1961).

### Subjective and objective measures of memory

To quantify subjective cognition, all participants completed the Cognitive Complaint Index battery (Saykin et al., 2006) to establish a cognitive complaint index (CCI) score. The CCI battery included the Memory Functioning Questionnaire (Gilewski et al., 1990), Memory Self-Rating Questionnaire (Squire et al., 1979), Neurobehavioral Function and Activities of Daily Living Rating Scale (Saykin, 1992), Informant Questionnaire on Cognitive Decline in the Elderly (IQCDE) (Jorm et al., 1994), 4 items related to cognition from the Geriatric Depression Scale (GDS, Yesavage et al., 1982), 12 items from a telephone-based screening for mild cognitive impairment (MCI), and 20 items from the Memory Assessment Questionnaire adapted in part from the Functional Activities Questionnaire. The CCI quantifies the degree to which women perceived their memory to be problematic (Saykin et al., 2006; Dumas et al., 2013). Responses to 114 questions were dichotomized as representing an endorsed or unendorsed complaint. The CCI score is expressed as the percent of all items endorsed. Participants were categorized in the cognitive complaint (CC) group if they endorsed more than 20% of the items on these questionnaires. Conversely, participants were categorized in the non-cognitive complaint (NC, *n* = 16) group if they endorsed less than 20% of items on the CCI.

The Selective Reminding Task (SRT, Buschke and Fuld, 1974) was used as the objective memory test. The SRT is a test of immediate and delayed episodic memory recall. Participants are read a list of 16 words and must immediately recall the list across 8 trials. Every trial after the first involves selectively reminding the participant of the words she did not recall on the immediately preceding trial. The SRT is continued until either the subject is able to correctly recall all 16 words on three consecutive trials, or until 8 trials have been completed. Upon completing the immediate recall portion of the SRT, and after a 20-min delay, participants are asked to complete a single delayed recall trial. SRT total immediate recall was analyzed using the number of correctly recalled words across trials 1–8, total immediate recall consistency was analyzed using the number of words correctly recalled on two trials in a row across trials 1–8, SRT total immediate recall failure was analyzed using the number of words not recalled two trials in a row across trials 1–8, and total delayed recall was analyzed using the number of words correctly recalled after a 20-min delay. Data were analyzed using SPSS to perform individual Pearson Correlation Tests between CCI scores and total immediate recall, total consistency, and total recall failure across trials 1–8. An additional correlation analysis compared CCI with total delayed recall.

### MRI image acquisition and pre-processing

At the University of Vermont and Vanderbilt, imaging data were collected using identical 3T Philips Achieva MRI (Philips Medical Systems, Inc., Best, Netherlands) scanners. Both scanners were identical in software and hardware. Scanner site has been included in the model for prior analyses using the imaging data collected at both sites and no significant effect was observed (Albert et al., 2015). Resting state fMRI was collected in the absence of external stimuli using an fMRI resting SENSE sequence (FOV = 240 mm^2^, matrix size = 80 × 80, 3 × 3 × 5 mm^3^ voxels, *TR* = 1500 ms, *TE* = 35 ms, flip angle = 90°, 0 mm gap, 5 mm slice thickness, 24 axial slices, 256 volumes). A high-resolution T1-weighted (T1W) fast field echo structural scan (FOV = 256 mm^2^, 1 mm isotropic voxels, TR = 9.8 ms, TE = 4.6 ms, flip angle = 8°, 140 sagittal slices) was collected to provide a template for image registration. The resting state scan was acquired after two cognitive and one emotion pictures task. All functional imaging data passed Vanderbilt in-house quality assurance evaluating signal-to-noise ratio, percent drift, percent fluctuation, radius of decorrelation, and percent standard deviation.

All functional images underwent quality assurance and standard preprocessing in SPM8 (Wellcome Department of Imaging Neuroscience, London, UK; http://www.fil.ion.ucl.ac.uk/spm/), which included slice-timing correction, motion correction, band-pass filtering (0.01 Hz < f < 0.1 Hz), T1W-EPI co-registration, spatial normalization, and spatial smoothing (6 mm FWHM). Individual T1W images were segmented into gray matter, white matter and cerebrospinal fluid tissue maps; then the T1W image, functional EPI and tissue maps were normalized to MNI152 space (Montreal Neurological Institute).

### Connectivity analysis

As previously described (Woodward et al., 2012; Vega et al., 2015), individual networks were identified per participant using the Conn toolbox v13 in SPM8, a Matlab-based functional connectivity toolbox (www.nitrc.org/projects/conn; Whitfield-Gabrieli and Nieto-Castanon, 2012). Each participant's normalized structural and functional images and T1W tissue maps were used as input into Conn. Importantly, the Conn toolbox first implements an anatomical, component-based, noise correction strategy (Compcor) to identify and reduce physiological and other noise signals that are unlikely to be related to neural activity (Whitfield-Gabrieli and Nieto-Castanon, 2012). After regressing out Compcor-identified noise, the resulting BOLD time series were band-pass filtered (0.008–0.09 Hz) to further reduce noise and increase sensitivity. The output matrices of SPM movement were entered into Conn as first-level covariates. Functional connectivity networks and their respective seeds used in the analysis are listed in Table 1. Seeds for each connectivity network were created using the WFU PickAtlas (http://www.fmri.wfubmc.edu/cms/software, version 2.3; Maldjian et al., 2003). The resting state networks listed in Table 1 were chosen because those networks have been associated with performance on cognitive tasks in previous studies (van den Heuvel and Hulshoff Pol, 2010). In addition to the resting state networks, seed regions (referred to as CCI-relevant regions) that showed structural and functional difference between cognitive complainers and control groups were also included in the analysis (Saykin et al., 2006; Wang et al., 2006, 2013; Dumas et al., 2013).

**Table 1:** **Functional connectivity networks and respective seeds**.

**Resting-state networks/ROIs**	**Seed region**	**MNI coordinates**
Executive Control Network (ECN)	Left dorsolateral prefrontal cortex	−42, 34, 20
	Right dorsolateral prefrontal cortex	46, 36, 18
Default Mode Network (DMN)	Posterior cingulate cortex	1, −55, 17
Dorsal Attention Network (DAN)	Left superior parietal lobule	−27, −52, 57
	Right superior parietal lobule	24, −56, 55
Salience Network	Left frontoinsular cortex	−32, 26, −14
	Right frontoinsular cortex	36, 26, −8
CCI-relevant regions	Right middle temporal gyrus	50, −8, −16
	Left middle frontal gyrus	−33, 42, 11

*List and coordinates of seed regions. Cognitive Complaint Index (CCI)-relevant areas refer to regions previously found to be distinguished based on subjective reporting of cognitive complaints, with higher BOLD activation during a working memory task and smaller volume*.

Group networks were identified in SPM8 for positive and negative connectivity to the seeds. Second level random effects analyses were used to create within group statistical parametric maps for each network seed ROI and to examine the relationship between connectivity and CCI score. For each network, the within group thresholded maps of positive correlation were combined across all participants to create a single mask containing voxels that positively (positive connectivity) or negatively (negative connectivity) correlated with the seed ROI at the a priori threshold. These were used to restrict the regression analysis to only those voxels that positively (positive connectivity) or negatively (negative connectivity) correlated with the respective network seeds. All statistical maps were thresholded at the cluster-level corrected alpha level (*p* = 0.05) for the voxel- wise *p* value (0.001).

## Results

### Relationship between demographics and CCI

Data are summarized in Table 2. Mean CCI and range of scores were: CC group mean CCI = 0.32, range = 0.20–0.51, NC group mean CCI = 0.10, range = 0.00–0.19. No significant correlations were found between CCI score and age, education, years since menopause, or measures of dementia (DRS and MMSE). CCI was positively correlated with the Menopause Symptom Checklist (*r*_(29)_ = 0.69, *p* < 0.01) and BDI (*r*_(29)_ = 0.49, *p* < 0.01). Items on the Menopause Symptom Checklist endorsed by more than 50% of participants were forgetfulness (71%), hot flashes (61.3%), sweet cravings (58.1%), early awakening (58.1%), joint pain (54.8%), and night sweats (51.6%). Items not endorsed by any participant were bleeding/spotting, decreased appetite, abdominal cramps, suffocation, panic attacks, fever, vomiting, breast sensitivity, heavy menstrual flow, and blind spots. Items on the BDI endorsed by more than 50% of participants were reduced interest in sex (83.9%), sleep (83.9%), tiredness (58.1%), and physical appearance (58.1%). Items not endorsed by any participant were failure, guilt, punishment, and suicidal thoughts. No differences were observed between CC and NC groups; CCI score was therefore analyzed as a continuous variable for the remainder of the analyses.

**Table 2:** **Demographic information and correlation with cognitive complaint index scores**.

	**Mean ± SD**	**Correlation with cognitive complaint index scores**
Age	56 ± 3.30	*r*_(29)_ = −0.13, *p* = 0.48
Education, no. years	16.13 ± 2.30	*r*_(29)_ = −0.26, *p* = 0.16
Years Since Menopause	7 ± 5.00	*r*_(29)_ = −0.6, *p* = 0.75
Menopause Symptom Checklist	17.35 ± 10.25	*r*_(29)_ = 0.69, *p* < 0.01^*^
Beck Depression Inventory (BDI)	2.9 ± 3.20	*r*_(29)_ = 0.49, *p* < 0.01^*^
Dementia Rating Scale (DRS)	140.9 ± 3.50	*r*_(29)_ = 0.21, *p* = 0.25
Mini-Mental State Examination (MMSE)	28.9 ± 1.20	*r*_(29)_ = −0.11, *p* = 0.56
Buschke SRT Total Immediate Recall	87.8 ± 14.50	*r*_(29)_ = 0.06, *p* = 0.75
Buschke SRT Total Recall Failure	8.9 ± 8.00	*r*_(29)_ = 0.1, *p* = 0.60
Buschke SRT Total Delayed Recall	10.4 ± 3.70	*r*_(29)_ = 0.04, *p* = 0.85

* *significance at p < 0.01. Data calculated for n = 31 participants*.

### Relationship between CCI and objective measures of memory

Several objective measures of cognition (Table 2), calculated from performance on the SRT, were found to be not correlated with CCI: immediate recall (*r* = 0.06, *p* > 0.7), recall failure (*r* = 0.1, *p* = 0.6), and delayed recall (*r* = 0.04, *p* > 0.8). In summary, healthy post-menopausal women who are in middle-age report a broad range of subjective cognitive complaints. Subjective cognitive complaints were not correlated with objective measures of memory, but were correlated with subjective reporting of other menopause- and age-related changes.

### Relationship between CCI and functional connectivity

**Figure 1:**
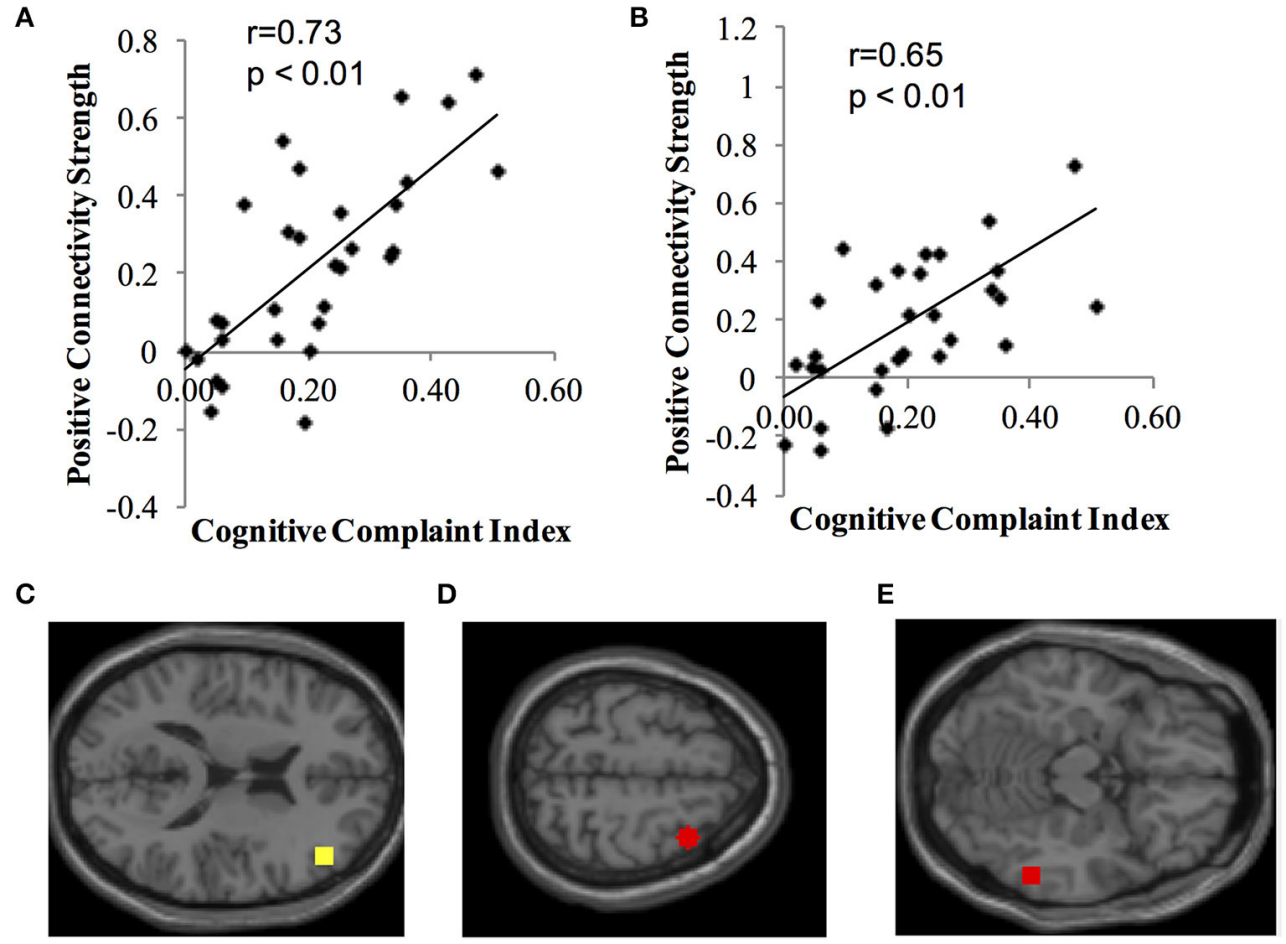
**Positive correlation between functional connectivity of the ECN and CCI. Data show positive connectivity**. **(A)** Y-axis depicts functional connectivity between the right seed region for the ECN (dorsolateral frontal cortex) and MNI 36, 10, 60 (right middle frontal gyrus). **(B)** Y-axis depicts functional connectivity between the right dorsolateral frontal cortex and MNI 58, −46, −14 (right temporal lobe, fusiform gyrus). **(C)** Yellow dot indicates the location of the seed region for the ECN, right dorsolateral prefrontal cortex. **(D)** Red dot indicates the location of the target region expressing functional connectivity with the dorsolateral prefrontal cortex in **(A)** (MNI 36, 10, 60; right middle frontal gyrus). **(E)** Red dot indicates the location of the target region expressing functional connectivity with the dorsolateral prefrontal cortex in **(B)** (MNI 58, −46, −14; right temporal lobe, fusiform gyrus).

Identified resting state networks for positive and negative connectivity to each network's seed ROI are depicted. Second level analysis revealed positive correlations (positive connectivity) between ECN seeds and CCI score (Figure 1). Participants who, subjectively, reported greater cognitive complaints demonstrated greater positive connectivity between the right dorsolateral prefrontal cortex seed ROI and the right middle frontal gyrus (MNI 36, 10, 60, cluster size = 91 voxels) and the right fusiform gyrus of the temporal lobe (MNI 58, −46, −14, cluster size = 51 voxels). There were no negative correlations between CCI and ECN, and no positive or negative correlations between CCI and DMN, DAN, or Salience Network seeds.

**Figure 2:**
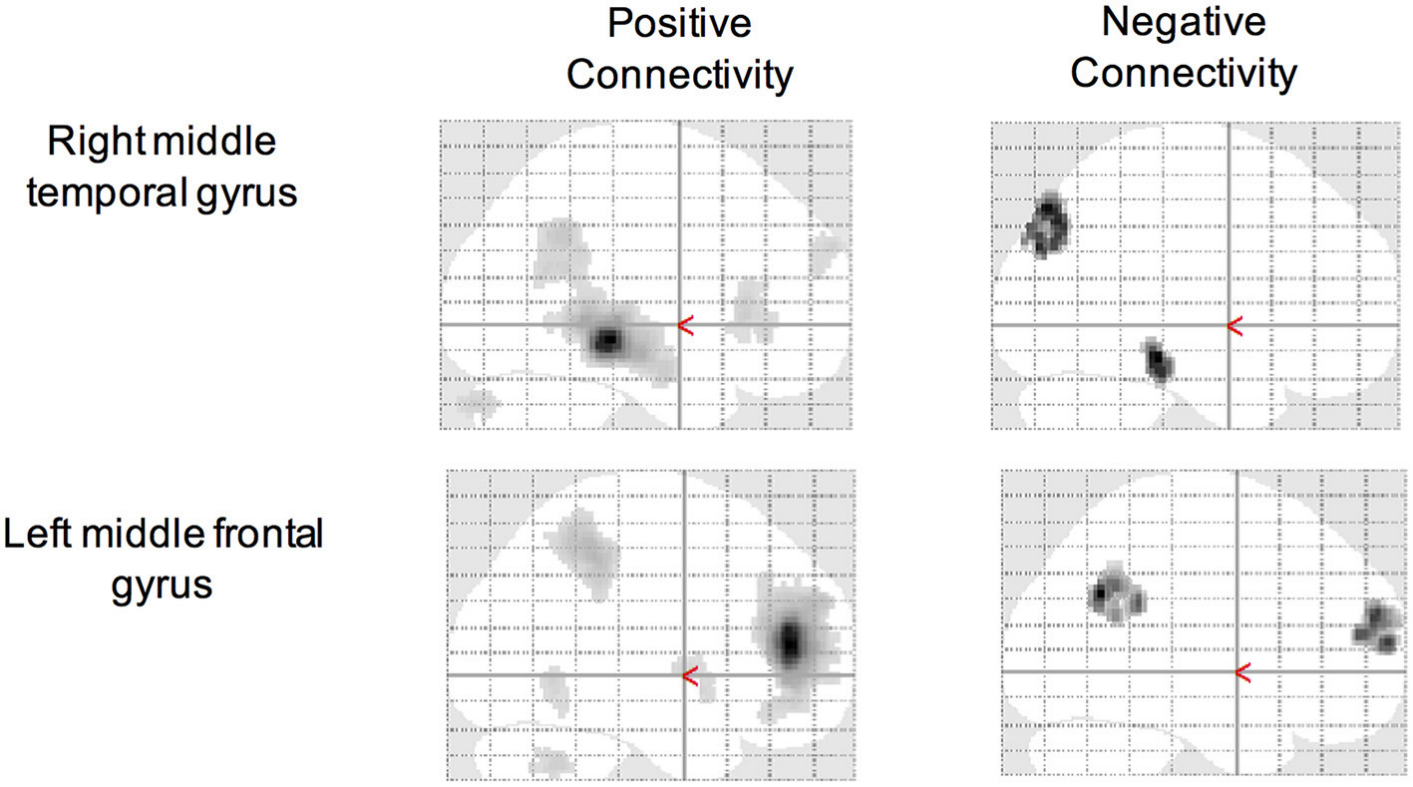
**Resting state networks of CCI-relevant regions**.

**Figure 3:**
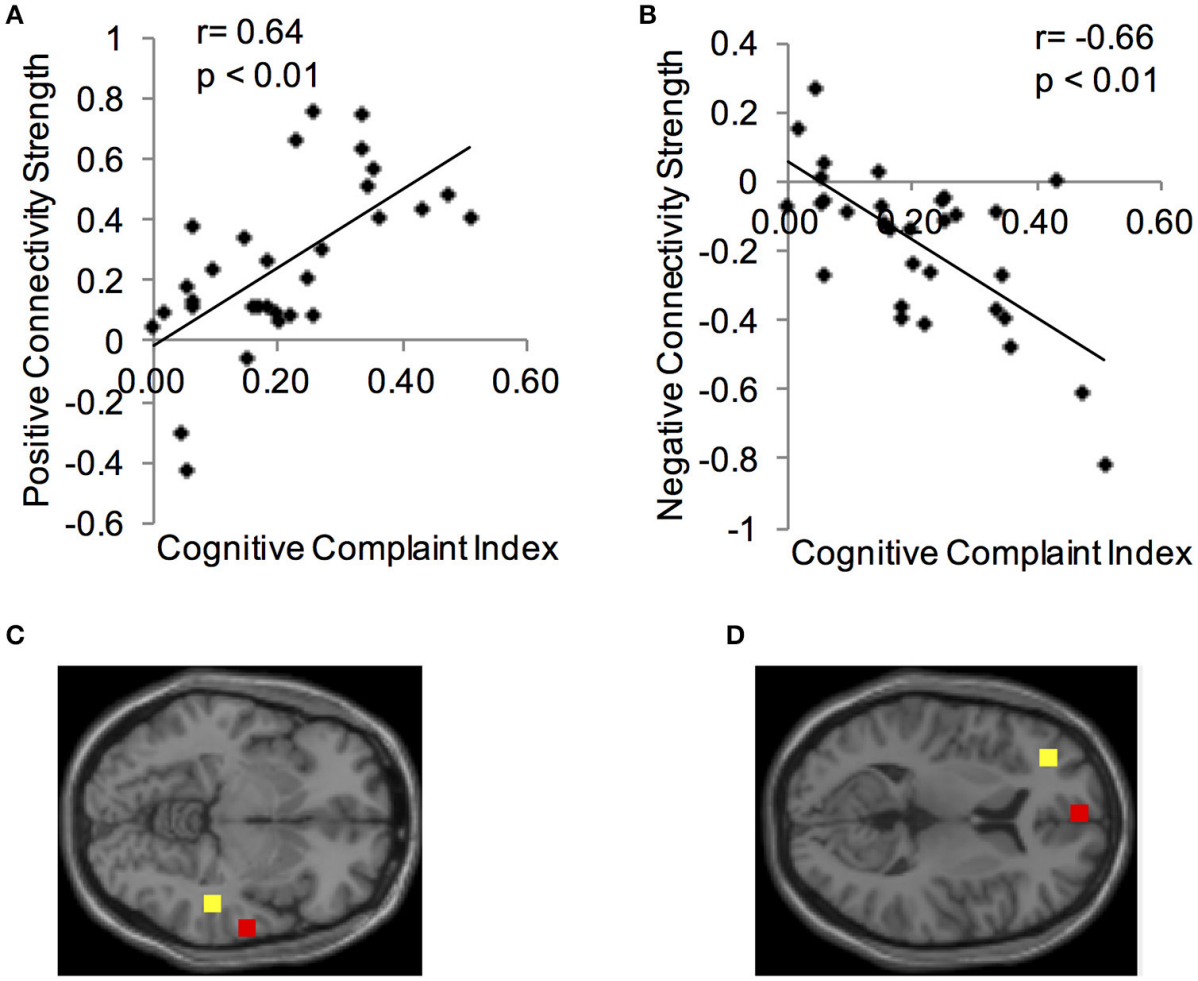
**Correlations between functional connectivity of a CCI-relevant region and Cognitive Complaint Index. (A)** Y-axis depicts positive functional connectivity between two regions of the right middle temporal gyrus. **(B)** Y-axis depicts negative functional connectivity between two regions of the left middle frontal gyrus. **(C)** Seed and target regions for connectivity strength in **(A)**. Yellow, seed region; red, target region (MNI 64, −12, −8). **(D)** Seed and target regions for connectivity strength in **(C)**. Yellow, seed region; red, target region (MNI −2, 58, 10).

Using the CCI-relevant regions as seed ROIs, functional connectivity networks were identified (Figure 2). As above, functional connectivity for positive and negative relationships to the CCI-relevant seed regions was calculated. Relationships were identified between CCI and two regions shown to be sensitive to CCI score in previous studies. Functional connectivity between two regions of the right middle temporal gyrus was positively correlated with CCI (target region MNI 64, −12, −8, cluster size = 36 voxels; Figures 3A,C). In the other direction, functional connectivity between two regions in the left middle frontal gyrus was negatively correlated with CCI (target region MNI −2, 58, 10, cluster size = 38; Figures 3B,D). In addition, we ran an analysis to see if there was any correlation between connectivity and BDI and menopause symptom checklist scores. Both greater depressive symptoms (*r* = −0.54, *p* = 0.002) and menopausal symptoms (*r* = −0.69, *p* < 0.001) were negatively correlated with negative connectivity with left middle frontal gyrus (CCI target region MNI −2, 58, 10, cluster size = 38). In summary, participants with higher CCI score had stronger positive functional connectivity within the right middle temporal gyrus, but also had weaker negative functional connectivity within the left middle frontal gyrus. Women with greater depressive symptoms and menopausal symptoms had weaker negative functional connectivity within the left middle frontal gyrus.

## Discussion

This study demonstrates relationships between rsFC and subjective cognitive complaints following menopause in otherwise cognitively normal, postmenopausal women. Specifically, higher CCI score was associated with greater functional connectivity in the ECN and within the temporal lobe, but reduced functional connectivity within the left frontal lobe. No differences were seen in other networks. We also observed that women with greater depressive symptoms and menopausal symptoms had weaker negative functional connectivity within the left middle frontal gyrus. It is possible that depressive symptoms and/or menopausal symptoms could be contributing to the weaker negative functional connectivity observed within the left middle frontal gyrus. However, both the BDI and menopause symptom checklist also contain items that evaluate cognition in addition to mood and somatic symptoms, therefore it is difficult to disentangle which symptoms are driving this effect. Previous work from our group has shown that in a subset of these participants, women with higher CCI scores had greater cortical activity in frontal and precuneus regions during a working memory task despite equivalent performance (Dumas et al., 2013). Dumas and colleagues concluded that this increased activation was a compensation response such that healthy middle-aged women with subjective cognitive complaints recruited a greater extent of the working memory network to maintain task performance. The increased resting state connectivity seen in this study may similarly reflect increased cognitive effort to maintain adequate performance for either normal or early pathologic cognitive aging processes. Although, we favor this hypothesis, we acknowledge the preliminary nature of these findings and the possibility that factors other than CCs mediate or contributes to the alterations in functional connectivity observed in this study. Future longitudinal studies examining connectivity during a task are needed to determine if increased connectivity does in fact reflect increased cognitive effort.

Increased connectivity has also been seen in populations at risk for the development of later life cognitive impairment (Filippini et al., 2009; Dennis et al., 2010; Sheline et al., 2010; Westlye et al., 2011). Our findings are consistent in some respects with those of Hafkemeijer and colleagues (Hafkemeijer et al., 2013), who found increased resting state functional connectivity in elderly normal adults with subjective memory complaints, although the predominant difference between the groups was found in the DMN and medial visual network rather than the ECN as was found in the current study. In the Hafkemeijer et al. study structural measures differed between the subjective complaint and control groups and correlated with increased functional connectivity (Hafkemeijer et al., 2013), however a preliminary structural analysis in the sample used for this study did not reveal substantial structural differences in gray matter volumes (unpublished data). Utilizing magnetoencephalography, Bajo and colleagues have also documented alterations in functional connectivity in healthy elderly subjects with increased subjective memory complaints compared to non-complaining elders and patients with MCI during the performance of a memory task (Bajo et al., 2012). Elders with subjective memory complaints showed lower functional connectivity in memory systems during task performance compared to cognitively normal and MCI patients (who showed increased synchronization), suggesting an initial decrease in functional connectivity, and later compensation through increased connectivity as cognitive difficulties progress from subjective to objective deficits.

Data from the Seattle Midlife Women's Health Study (Woods et al., 2000) suggest that nearly half of postmenopausal women report noticeable cognitive symptoms, including attention, concentration, and memory problems. Approximately a third of women reported these problems as at least moderate in severity. Subjective cognitive complaints in the postmenopausal period may be a useful index of developing cognitive and/or brain dysfunction. Older adults with subjective cognitive complaints but normal cognitive performance have been shown to convert to dementia at higher rates than those without complaints (Reisberg et al., 2010) and have structural and functional changes in the brain that may indicate developing neurodegenerative disorders (Saykin et al., 2006; Rodda et al., 2009; Lamar et al., 2011). Cognitive complaints have also been associated with the menopause transition (Weber and Mapstone, 2009) and related to poorer working memory and encoding performance, while data showing objective cognitive impairments after menopause are inconsistent (Hogervorst and Bandelow, 2010).

Women who notice significant cognitive disruption following menopause may be vulnerable to disruption of cholinergic or other brain systems due to lower cognitive reserve, early neurodegeneration, or other factors. Thus, the loss of estrogen support at menopause may be more noticeable in terms of cognitive processes, particularly attention, executive function, and verbal memory in these vulnerable women. Cognitive complaints or changes in cognitive performance after menopause may be an important indicator of risk for late life cognitive impairment. Accumulating evidence suggests that subjective cognitive complaints have significant predictive value in assessing the risk of development of dementia in elderly individuals (Jessen et al., 2014b). Whether such complaints in the first few years after menopause can be similarly used to identify those at increased risk for cognitive dysfunction or dementia in late life will require longer follow-up, but early changes in brain connectivity as shown in this study or changes in task-related cortical activity (Dumas et al., 2013) suggest that the neural manifestations of early cognitive change may be apparent many years prior to the development of measurable cognitive impairment. Information from longitudinal brain aging studies has suggested that it is possible to identify a subgroup of otherwise normal individuals in middle-age who show evidence of pathologic brain changes (e.g., increased beta amyloid deposition) who may be expected to be at elevated risk for the development of AD later in life (Rodrigue et al., 2012). Previous studies indicate that in cognitively normal older adults, amyloid burden is increased in individuals with cognitive complaints (Amariglio et al., 2012; Perrotin et al., 2012).

Successful decision-making and action depend on accurately evaluating the success of basic cognitive processes that contribute to thought and behavior, a capacity known as “metacognition” (Metcalfe and Shimamura, 1994). Memory self-efficacy can be regarded as an aspect of metamemory or metacognition. Subjective memory complainers have a lower rating of their own memory capacity and therefore a lower memory-related self-efficacy than people without subjective memory complaints (Ponds et al., 1992; Ponds and Jolles, 1996). Furthermore, low memory self-efficacy is a key features of memory complainers (Metternich et al., 2009). The current findings suggest that subjective cognitive complainers may have some impairment in metacognition. Medial and lateral networks in anterior prefrontal cortex (aPFC) support metacognitive ability for memory and perception (Baird et al., 2013). BOLD signal in right posterior-lateral BA10 was positively correlated with metacognitive accuracy (Yokoyama et al., 2010). Convergent evidence indicates that frontopolar Brodmann area 10, and more generally the aPFC, support the human capacity to monitor and reflect on cognition and experience (Fleming and Dolan, 2012; Baird et al., 2013). Prefrontal cortex integrity seems to play an essential role in metacognitive judgments. The negative correlation between connectivity in the left middle frontal gyrus and CCI in this study may indicate that metacognitive impairments in subjective cognitive complainers are related to decreased connectivity with aPFC. Impairment of prefrontal cortex integrity may lead to metacognitive dysfunction, in response to which subjective cognitive complainers may use more neural resources to compensate than non-complainers (Dumas et al., 2013). This compensation explanation may be relevant to the finding of increased functional connectivity within the medial temporal lobe in MCI (Das et al., 2013).

Since the loss of estradiol is the critical hormonal event with impact on brain function that occurs at menopause, the question of whether a period of hormonal treatment could alter either the cognitive complaints or the neural representation of the cognitive complaints (increased connectivity and task-related activity) deserves further study. The “critical period hypothesis” (Resnick and Henderson, 2002; Maki, 2006; Sherwin, 2006, 2007) suggests that estradiol has maximal protective benefits on cognition in women when it is initiated proximal to the menopause but may be ineffective when initiated many years or decades later. What has not been clearly established is whether there is a subgroup of women for whom postmenopausal hormone treatment has more long-term benefits on cognition. Whether individuals with cognitive complaints following menopause are at higher risk for age-related cognitive decline and/or cholinergic dysfunction and would benefit from postmenopausal estradiol treatment will require further study.

## Author contributions

PN, BM, AS, MN, and JD, contributed to the conception or design of the work; KA, LZ, contributed to data collection; LZ, KA, BB, NW, JV, JD, PN AM, conducted the data analysis and/or contributed to interpretation of the data; JV, LZ, drafted the article; JV, KA, PN, BM, AS, JD, KA, and JP, contributed to critical revision of the article, and all authors gave final approval of the version to be published.

### Conflict of interest statement

The authors declare that the research was conducted in the absence of any commercial or financial relationships that could be construed as a potential conflict of interest.

## Abbreviations

SCD: subjective cognitive decline
rsFC: resting state functional connectivity
DMN: default mode network
DAN: dorsal attention network
VAN: ventral attention network
ECN: executive control network
CCI: cognitive complaint index
CC: cognitive complainer group
NC: non-complainer group
SRT: selective reminding task.
